# Burkitt lymphoma after adult liver transplantation: a case report and literature review

**DOI:** 10.3389/fonc.2024.1439137

**Published:** 2024-12-10

**Authors:** Ji Zhang, Qing Chen, Shuhua Zhang

**Affiliations:** Department of Hepatobiliary Surgery of General Surgery, Union Hospital, Tongji Medical College, Huazhong University of Science and Technology, Wuhan, China

**Keywords:** Burkitt lymphoma (bl), liver transplantation, adult, case report, review

## Abstract

**Preface and importance:**

Burkitt’s lymphoma (BL) is a relatively rare post-transplant lymphoproliferative disorder (PTLD), and there is currently limited research on the occurrence of BL following adult liver transplantation.

**Case introduction:**

We report a 45-year-old male who developed BL that rapidly progressed at seven years after left liver transplantation. The patient eventually abandoned treatment due to severe clinical complications.

**Clinical discussion:**

BL is a highly invasive B-cell-derived non-Hodgkin lymphoma (NHL), with fast progression and easy involvement of the central nervous system. The current case had sporadic BL with atypical site of onset. After analysis, the investigators considered the possible development of tumor lysis syndrome (TLS) in the later stage of hospitalization, which is a more serious complication of BL. There is currently no unified treatment plan for adult BL.

**Conclusion:**

BL is a relatively rare complication after liver transplantation, and its early detection and treatment are crucial. For advanced BL, attention should be given in preventing the occurrence of TLS. Further research and exploration are needed to determine the optimal treatment plan for adult BL.

## 1 Introduction

Post-transplant lymphoproliferative disorder (PTLD) is a relatively rare complication after organ transplantation. The incidence of PTLD after solid organ transplantation ranges within 1-10%, while the incidence of PTLD in adult liver transplant recipients range within 2-4% (1). PTLD often manifests as lymphoma associated with immunosuppression and Epstein Barr virus (EBV) after transplantation (2), and Burkitt’s lymphoma (BL) is one of the rarer types. BL is a highly invasive B-cell-derived non-Hodgkin lymphoma (NHL) that originates from mature, germinal, or post-germinal B cells (3). The current study reviews and analyzes the medical records of a patient who developed BL after liver transplantation, and was admitted to Tongji Medical College Affiliated Union Hospital of Huazhong University of Science and Technology in July 2022. In addition, the relevant literature was reviewed to provide references for clinical practice.

## 2 Clinical data

A 45-year-old male patient complained of “back pain for 30 days, weakness in both lower limbs for 15 days, and worsening for six days”, accompanied by acute urinary retention and paraplegia. The patient was admitted to the Neurosurgery Department of Tongji Medical College Affiliated Union Hospital (Wuhan Union Hospital) of Huazhong University of Science and Technology on July 19, 2022. The patient has a history of chronic hepatitis B and cirrhosis for nine years, and a history of renal insufficiency for three years. In July 2015, the patient underwent “orthotopic auxiliary liver transplantation with left lobe donor” in the Department of Hepatobiliary Surgery of Wuhan Union Medical College Hospital due to “decompensated hepatitis B cirrhosis with esophageal and gastric variceal bleeding”. The donor came from the patient’s father, and the patient regularly used immunosuppressant tacrolimus after discharge. In 2016, due to severe gastrointestinal bleeding, the patient underwent splenectomy and pericardial vessel dissection at the Hepatobiliary Department of Wuhan Union Medical College Hospital. Specialized physical examination: normal muscle tone in both upper limbs, high muscle tone in both lower limbs, grade I muscle strength, disappearance of lower limb physiological reflexes, and no pathological reflexes were introduced.

**Figure 1:**
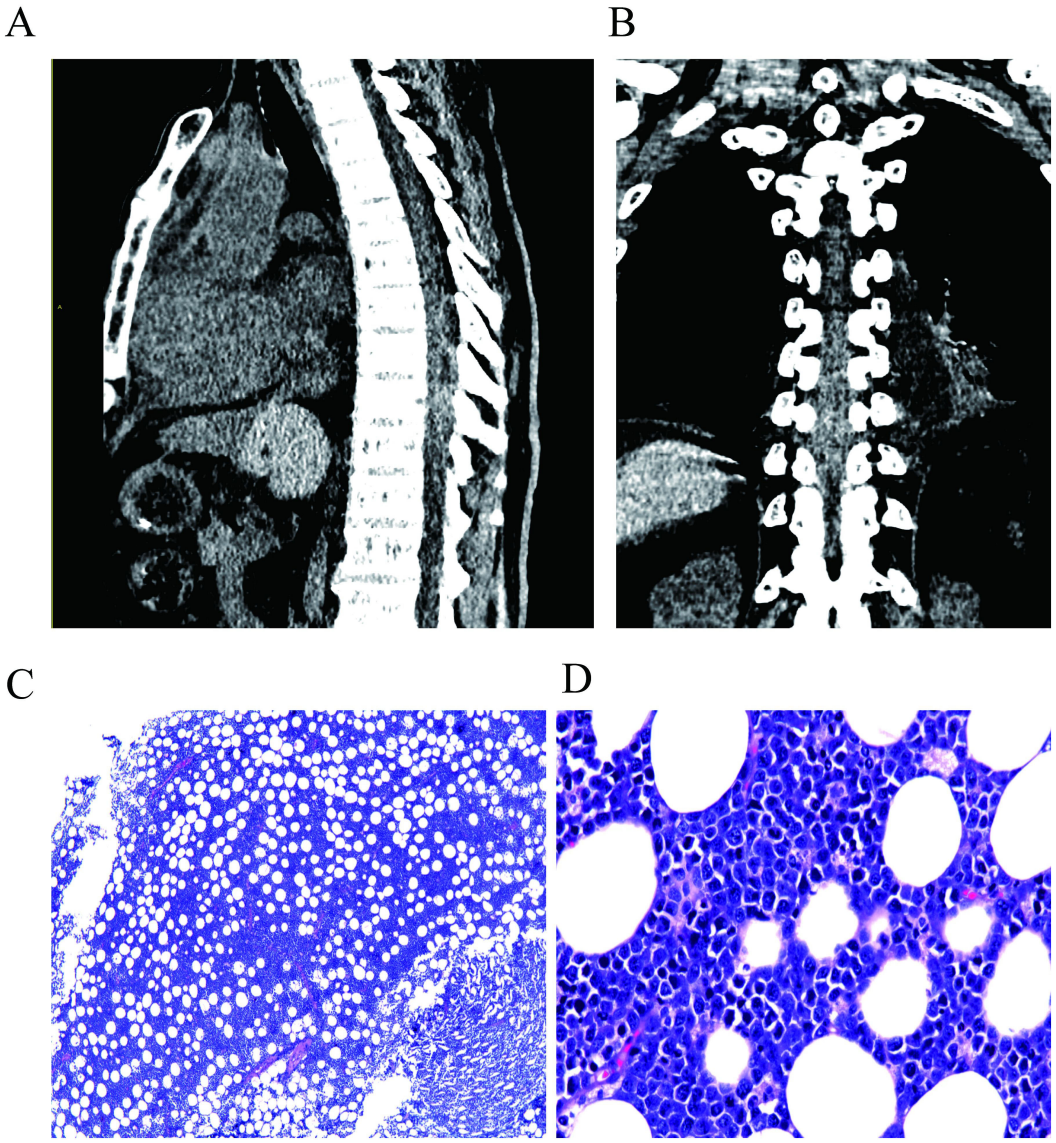
Preoperative CT scan of the patient, indicating a suspected epidural hematoma or mass lesion at the T7-T10 thoracic spinal segments. **(A)** shows the lateral view, while **(B)** shows the anterior view. The postoperative pathological image of the patient. **(C, D)** shows the images for the H&E staining at 40× and 400× magnification, respectively. Microscopically, medium-sized tumor cells were diffusely infiltrated, with vacuoles and round nuclei in the cytoplasm, and the cytoplasm was basophilic.

On July 18, 2022, our hospital’s emergency computed tomography (CT) results revealed a faint spindle shaped or slightly high-density shadow in the posterior part of the thoracic spinal canal at 7-10 segments, with unclear boundaries and a range of approximately 97 mm. Furthermore, the dural sac was pushed forward, and the CT value on plain scan was approximately 30-50 Hu, indicating suspected epidural hematoma or space occupying lesion (**Figure 1**). Laboratory examination: Blood routine test results: hemoglobin, 105 g/L (reference value: 130-175 g/L); white blood cell count, 14.31 G/L (3.5-9.5 G/L); platelet count, 235 G/L (125-350 G/L). Hepatitis B-related indicators: hepatitis B surface antigen, >250.0 IU/ml (<0.05 IU/ml); hepatitis B core antibody, 5.62 (+). Biochemical indicators: total bilirubin, 20.2 μmol/L (3.0-22.0 μmol/L); alanine aminotransferase, 22 U/L (<50 U/L); aspartate aminotransferase 36 U/L (17-59 U/L); γ-glutamyl transferase, 15 U/L (15-73 U/L); total protein, 47.0 g/L (63.0-82.0 g/L); albumin, 22.7 g/L (35.0-50.0 g/L); globulin, 24.3 g/L (23.0-32.0 g/L); urea nitrogen, 15.71 mmol/L (3.20-7.10 mmol/L); creatinine (enzymatic method), 133.6 μmol/L (58.0-110.0 μmol/L); uric acid, 442.1 μmol/L (208.0-506.0 μmol/L); sodium 135.3 mmol/L (137.0-145.0 mmol/L); potassium, 4.98 mmol/L (3.50-5.10 mmol/L); chlorine, 103.8 mmol/L (98.0-107.0 mmol/L); calcium 2.06 mmol/L (2.10-2.55 mmol/L). Cardiac-related indicators: creatine kinase, 261 U/L (38-174 U/L); lactate dehydrogenase, 1,207 U/L (109-245 U/L). Coagulation-related indicators: prothrombin time (PT), 16.3 seconds (11.0-16.0 seconds); activated partial thromboplastin time (APTT), 34.6 seconds (28.0-43.5 seconds); thrombin time (TT), 16.8 seconds (14.0-21.0 seconds); fibrinogen (FIB), 1.84 g/l (2.00-4.00 g/l).

On July 19, 2022, an emergency procedure of thoracic spine pedicle fixation with resection of intraspinal lesions (thoracic spine) was performed for the patient. The tumor was separated, and the lesion was completely removed, followed by nerve decompression (postoperative image). The excised tissue was identified as a pile of gray, red and yellow fragmented tissues (5 × 4 × 2 cm) that contained a small amount of bone. The bone was 9.0 × 3.0 × 1.5 cm without plastic surgery, and the cut surface was dark red. The postoperative immunohistochemical staining of tumor cells revealed the following: CD3 (-), CD5 (-), CD10 (+), CD19 (+), CD20 (partially+), CD21 (-), CD22 (partially+), CD30 (-), CD34 (-), CD38 (weakly+), CD43 (+), CD79a (+), CD138 (-), PAX5 (+), BCL6 (-), MUM1 (+), BCL2 (-), cyclinD1 (-), TdT (-), MPO (-), S100 (-). PCK (-), C-Myc (90%+), P53 (90% strong+), TCL1 (+), ERG (-), and Ki67 (LI: 90%). *In situ* hybridization detection of EBV: EBER (-). Fluorescence *in situ* hybridization (FISH) results: BCL-6 gene translocation (-), BCL-2 gene translocation (-), C-MYC gene translocation (+), and C-MYC/IgH gene fusion (-). Pathological examination results (**Figure 1**): (6-10 thoracic vertebrae epidural) highly invasive B-cell NHL, with tumor tissue involving the medullary cavity (6-10 thoracic vertebrae), combined with FISH detection results, consistent with BL, and classified as stage IV, according to the International Lymphoma Ann Arbor staging.

**Figure 2:**
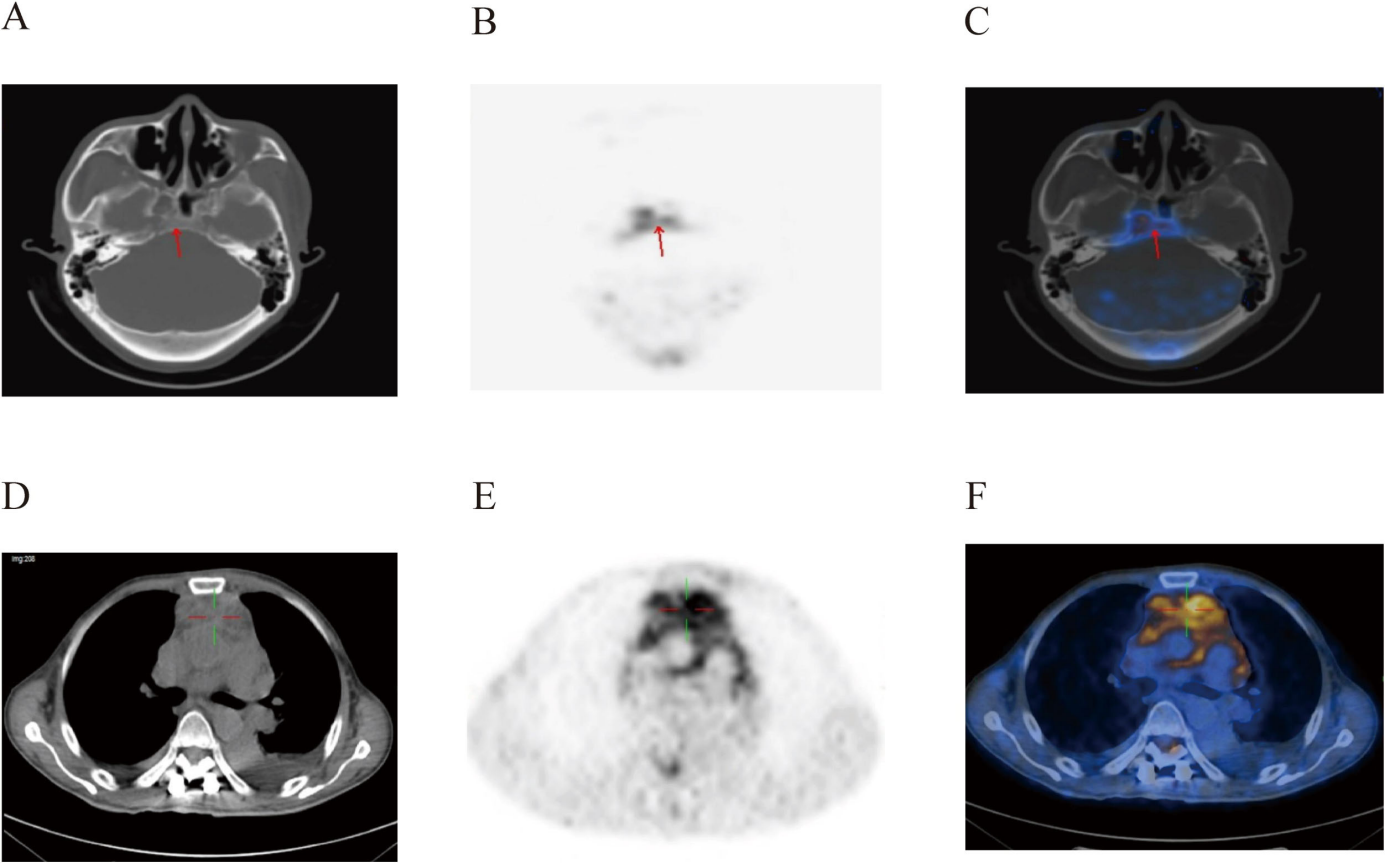
PET-CT images of the patient’s brain **(A–C)** and mediastinum **(D–F)** at post-surgery. There was an abnormal increase in metabolism in soft tissues of the nasopharynx and mediastinum.

**Figure 3:**
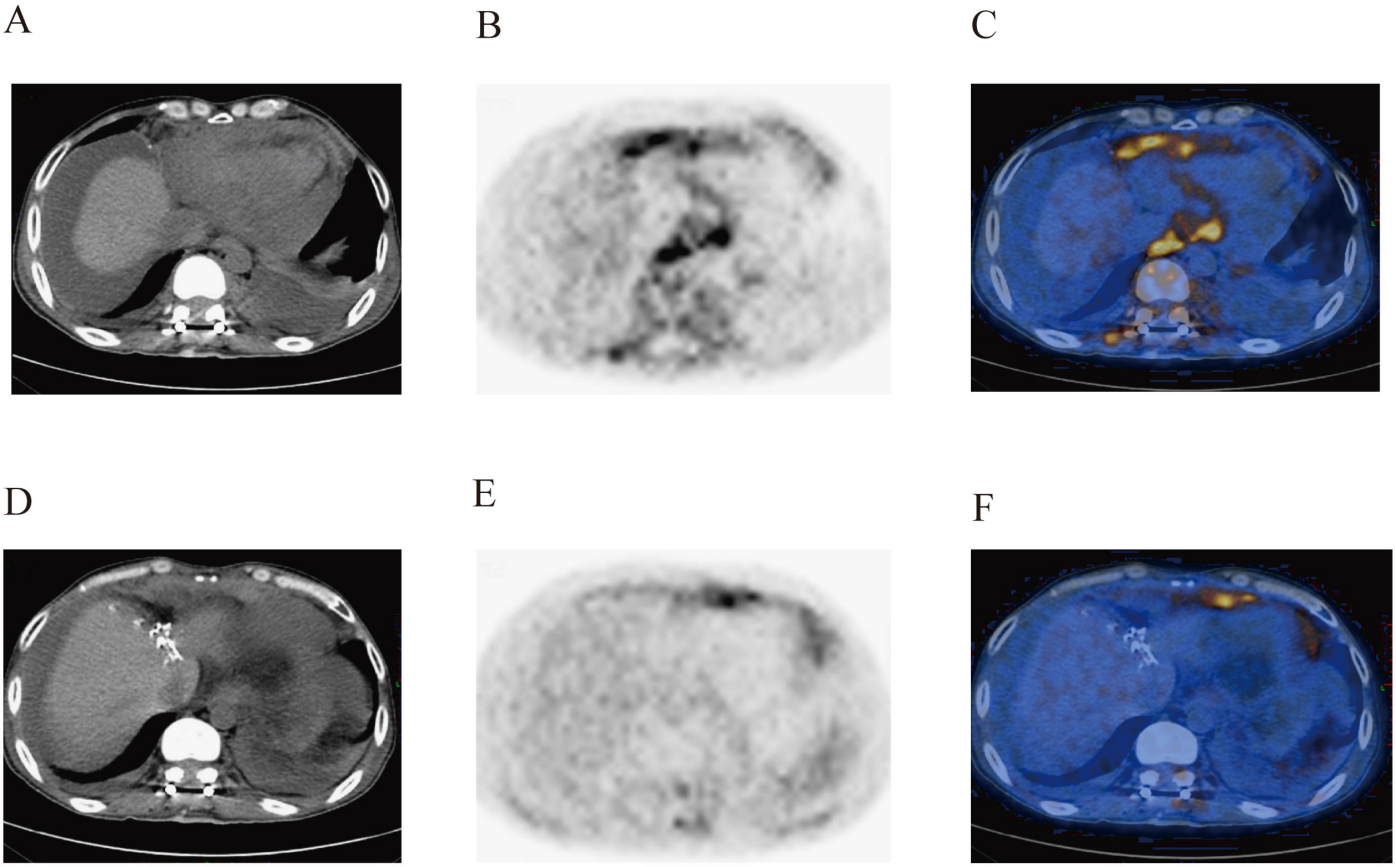
Postoperative PET-CT images of the patient’s vertebral body **(A–C)** and abdominal cavity **(D–F)**. The images show the abnormal increase in metabolism in the vertebral body, paravertebral soft tissues, abdominal visceral organs, and related tissues.

**Figure 4:**
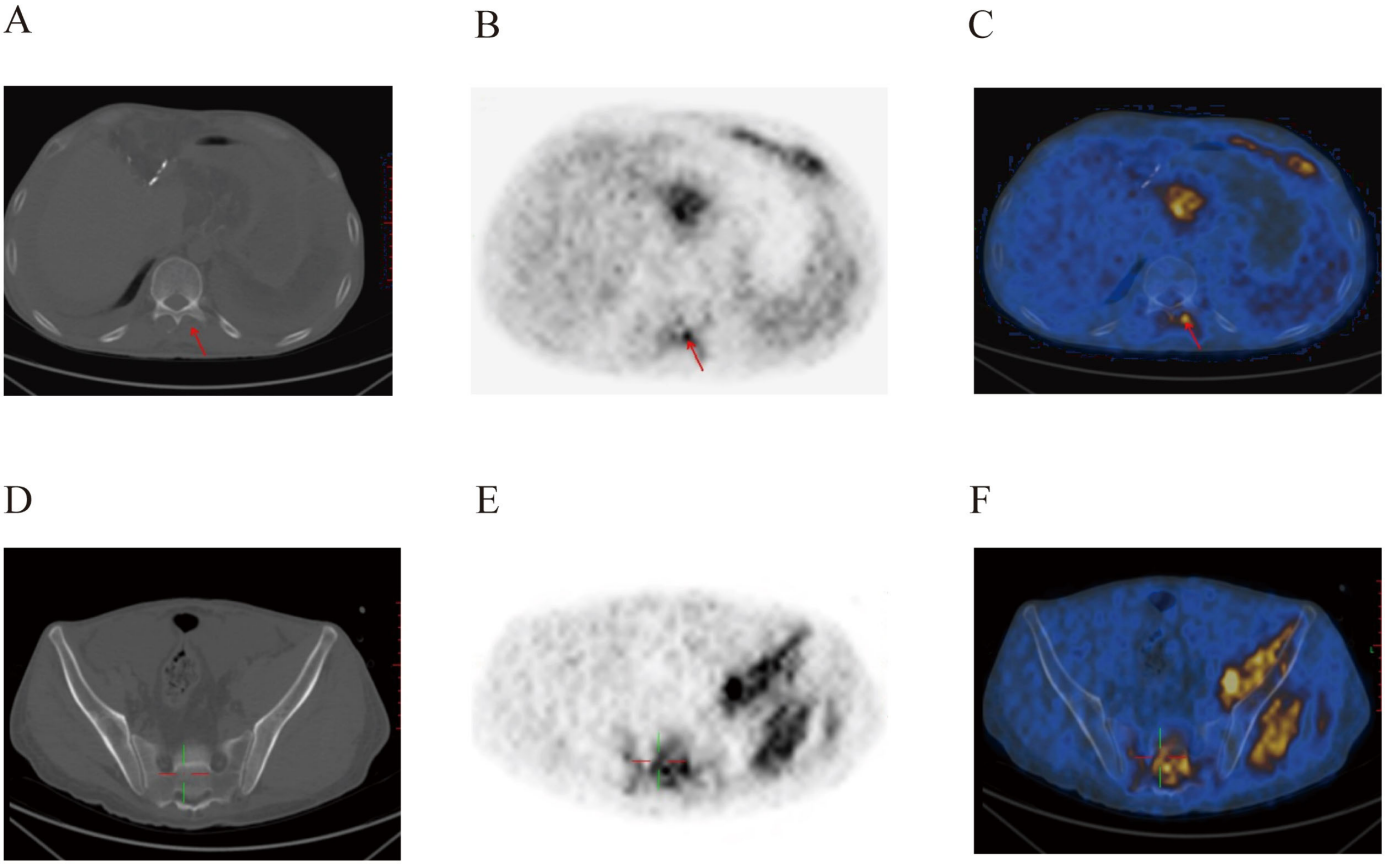
Postoperative PET-CT images of the patient’s spinal canal **(A–C)** and pelvic region **(D–F)**. Metabolic abnormalities were evident within the spinal canal, and in the left hip muscle.

On August 9, 2022, the patient underwent postoperative whole-body glucose metabolism positron emission tomography and computed tomography imaging (PET-CT), which revealed the following: (1) a mediastinal soft tissue mass with unclear boundaries, surrounding adjacent blood vessels, invading the pericardium and diaphragm, and with abnormally elevated metabolism; (2) the ascending colon had a thickened intestinal wall, an abnormally elevated metabolism, multiple lymph nodes in the hepatogastric space, retroperitoneum and mesangial area, partial fusion, and an abnormally elevated metabolism; (3) the adipose space in the abdominal cavity was cloudy, with thickened peritoneum, omentum and mesentery, and abnormal metabolic elevation; (4) suspected thickening of soft tissues and abnormal elevation of metabolism in the space between the lost sinus and sigmoid sinus; (5) the bone density of the right eyebrow arch, parietal bone, occipital bone and clivus, sphenoid body, and bilateral petrous apex were slightly uneven, and the metabolism was abnormally elevated; (6) abnormal elevation of metabolism in the T11-L1 attachment, L3 vertebral body, sacrum, left ilium, left ischium, and right femur; (8) abnormal increase in metabolism within the T3-T10 spinal canal, and abnormal elevation of metabolism in the left iliac muscle, left thoracic spine muscle, left gluteus medius muscle, and left gluteus maximus muscle; (9) the above conforms to the signs of malignant lymphoma infiltration; (10) thickening of soft tissues in the nasopharynx (on the right side), bilateral tonsils enlargement, and abnormal metabolic elevation, suggesting a high possibility of lymphoma infiltration (**Figures 2**–**4**).

**Figure 5:**
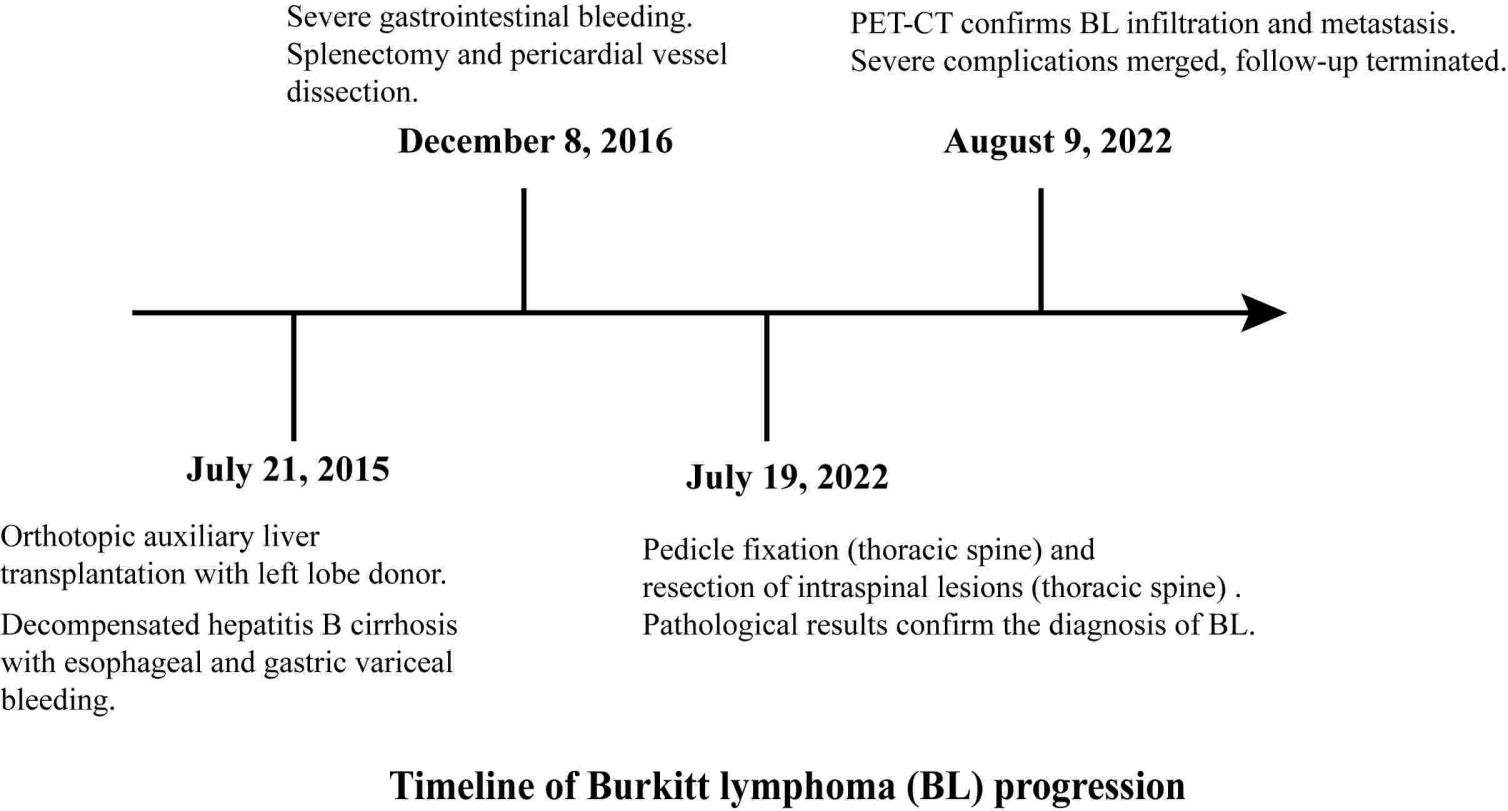
Timeline of Burkitt lymphoma (BL) progression.

**Figure 6:**
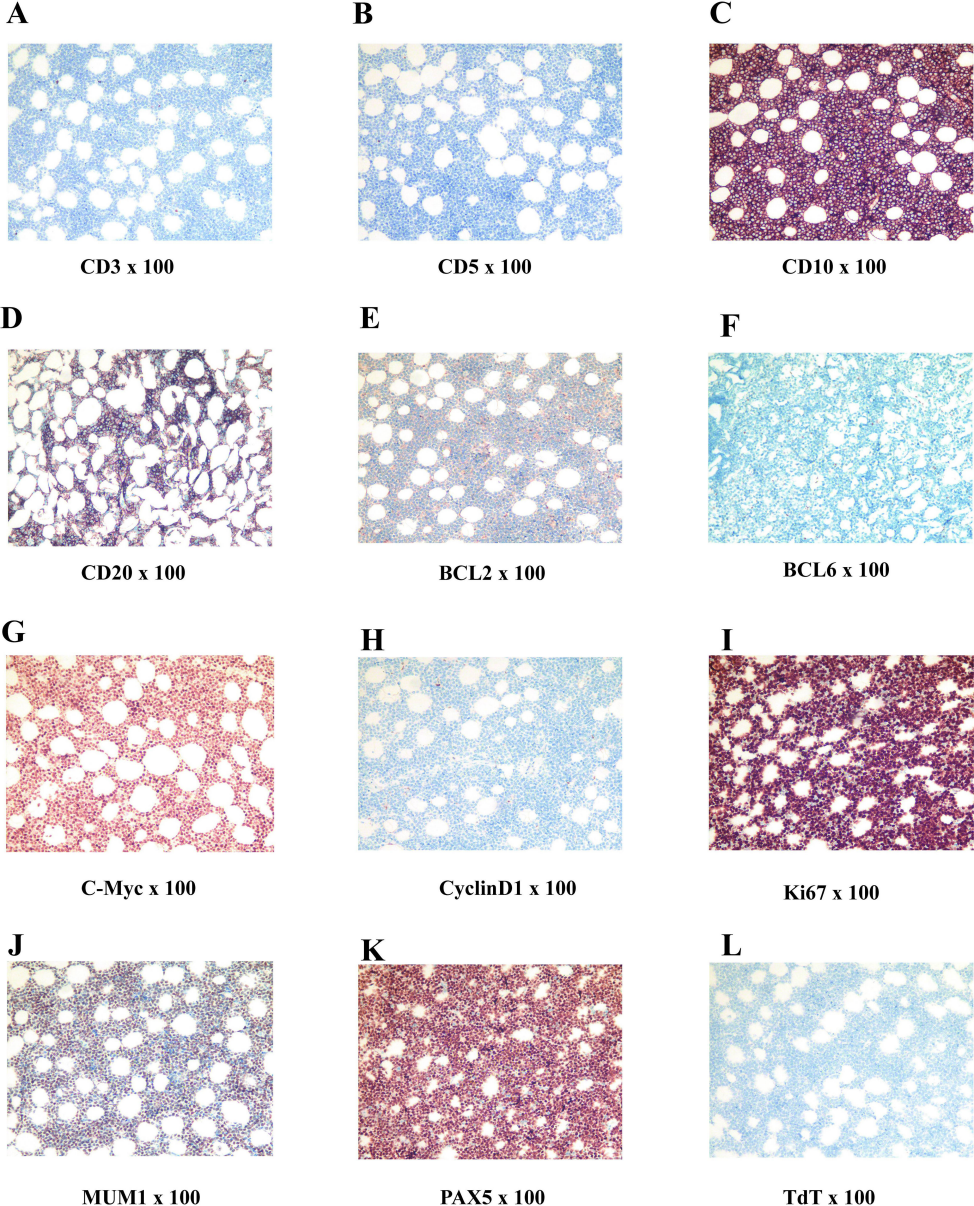
Postoperative immunohistochemistry related images of Burkitt lymphoma **(A–L)**.

On August 9, 2022, the relevant condition of the patient was extremely poor. The laboratory test results and biochemical indicators were, as follows: total bilirubin, 31.4 μmol/L (5.1-19.0 μmol/L); urea nitrogen, 29.27 mmol/L (2.90-8.20 mmol/L); creatinine (enzymatic method), 136.2 μmol/L (44.0-133.0 μmol/L); uric acid, 658.0 μmol/L (208.0-428.0 μmol/L); potassium, 4.75 mmol/L (3.50-5.20 mmol/L); calcium, 2.25 mmol/L (2.03-2.54 mmol/L). Cardiac-related indicators: creatine kinase, 150 U/L (38-174 U/L); lactate dehydrogenase, 3,411 U/L (109-245 U/L); creatine kinase isoenzyme CK-MB, 14.0 ng/ml (<6.6 ng/ml); hypersensitive troponin I, 2,322.9 ng/L (<26.2 ng/L). Coagulation-related indicators: PT, 26.6 seconds (11.0-16.0 seconds); APTT, 58.5 seconds (28.0-43.5 seconds); TT, 29.2 seconds (14.0-21.0 seconds); FIB, <0.6 g/l (2.0-4.0 g/l); fibrin degradation product (FDP), 21.9 ug/ml (<5.0 ug/ml); D-dimer, 6.63 mg/L (<0.50 mg/L); plasma antithrombin III (AT-III), 20% (80-120%). The patient’s coagulation function progressively decreased, reaching the diagnostic criteria for disseminated intravascular coagulation (DIC). In addition, BL in the advanced stage and complications, such as severe heart and kidney dysfunction, and thrombocytopenia, posed a life-threatening risk to the patient at any time. After consultation with the family, the patient was discharged on the same day, and no further follow-ups were conducted (**Figures 5**, **6**).

## 3 Discussion

PTLD is a relatively rare complication after hematopoietic stem cell transplantation or solid organ transplantation, and often manifests as lymphoma associated with immune suppression and EBV after transplantation. In adults, the occurrence time of PTLD after liver transplantation has two peaks. The first peak occurs at the first year after transplantation, mostly in EBV positive transplant recipients, while the second peak occurs at 5-15 years after transplantation, usually involving EBV negative receptors (2). For the present case, the patient developed PTLD at seven years after liver transplantation, which manifested as BL. The EBV-related test was negative, which is basically consistent with the above details.

BL is a highly invasive NHL (4). In the latest revised classification of lymphoid tumors in 2016, the World Health Organization classified BL into three different subtypes, namely, endemic, immunodeficiency-related, and sporadic (5). There are certain differences in epidemiology, clinical manifestations, and genetic characteristics among these above subtypes. For example, endemic BL is more common in children and adolescents in sub-Saharan Africa, and is generally associated with EBV infection (6). The clinical manifestations typically include rapidly expanding masses in the jawbone or periorbital region, which can also affect other organs, such as the spleen and gastrointestinal tract, while bone marrow and central nervous system (CNS) involvement are not common. Immunodeficiency-related BL is closely correlated to human immunodeficiency virus (HIV) infection, with clinical manifestations mainly involving enlarged lymph nodes, and this may also involve the bone marrow and CNS (4). Cases of BL have been reported worldwide, and BL is more common in children, accounting for approximately 20-30% of lymphomas. However, BL is rare in adults, and mainly manifests in male patients, with a male-to-female ratio of 2-3:1. Among these cases, EBV-related cases account for approximately 25-40% (7, 8).

For the current case, the patient was considered sporadic. The most common primary site of sporadic BL was in the abdomen, followed by the head and neck. In clinical practice, abdominal pain and gastrointestinal bleeding are often the first symptoms (9). Sporadic BL often easily affects the CNS, with common locations including the pia mater and bone marrow (10). BL can grow rapidly, and quickly spread to the extranodal area, with 70% of patients diagnosed as stage III or IV. Late staging often indicates poor prognosis. For the current case, the first site of the patient was in the thoracic vertebrae (T6-T10), which is not typical. The initial suspected symptoms were epidural hematoma or space occupying lesion (thoracic vertebrae), urinary retention, and paraplegia, indicating that the CNS was invaded. The postoperative pathology and PET-CT confirmed that the tumor invaded the spinal cord with multiple metastases throughout the body. The Ann Arbor stage of lymphoma was IV, which is a late disease stage.

In the immunohistochemistry and pathology, the histological features of BL presented with complete disappearance of lymph node structure, and generally medium size tumor cells, without pleomorphism, but contained basophilic cytoplasm, and small vacuoles and circular nuclei in the cytoplasm, as well as granular nuclear chromatin, small nucleoli, and frequent mitosis (8). A number of large and irregular macrophages in BL engulfed the apoptotic tumor cells, and was distributed among the lymphocytes, presenting a classic starry appearance. The high expression and translocation of the *MYC* gene can be observed in almost all subtypes of BL, which is a typical feature of BL. Ki-67 serves as an indicator of growth fraction in BL, and its expression is usually close to 100% (for the current case, the expression was 90%), indicating active tumor cell proliferation. BL generally express B cell markers, such as CD19, CD20, CD22 and CD79a. CD20 and CD79a positive cells usually express germinal center-related markers CD10 and BCL-6, but generally do not express BCL-2 and terminal deoxynucleotide transferase (TdT) (11, 12). In cytogenetics and molecular biology, 80% of BL cases exhibit mutual translocation between the *MYC* gene on chromosome 8 and the immunoglobulin heavy chain (IGH) gene (t [8; 14]) on chromosome 14. The translocation of MYC to immunoglobulin heavy chain or light chain genes can lead to the activation and overexpression of MYC components (4, 9), causing uncontrolled cell growth and proliferation.

In the diagnosis of BL, the clinical manifestations of BL may not be specific. When BL is highly suspected in clinical practice, in addition to conventional imaging methods and laboratory tests, resection or biopsy of the lesion site is generally a powerful means of diagnosis. In addition, for the involvement of the CNS, this can be evaluated through the cerebrospinal fluid puncture cytology results. Meanwhile, studies have revealed that the application of positron emission tomography (PET) or PET-CT can improve the accuracy of diagnosis and related staging (13). In the differential diagnosis, BL needs to be differentiated from other high-grade NHLs, including high-grade B-cell lymphoma, with both MYC and BCL-2, and/or BCL-6 translocations, as well as diffuse large B-cell lymphoma (DLBCL) with MYC translocation. Although these lymphomas may have overlapping histological and cytogenetic features with BL (12), these exhibit some differences. For example, DLBCL often express less CD30 and CD5, and histologically exhibits more heterogeneity, larger cells, and richer cytoplasm.

It is noteworthy that due to the faster proliferation rate and shorter doubling time of BL, late-stage BL patients often present with higher levels of lactate dehydrogenase (LDH), which requires vigilance against the occurrence of spontaneous tumor lysis syndrome (TLS). TLS is the result of the release of intracellular substances into the bloodstream, and is often characterized by hyperkalemia, hyperphosphatemia, hypocalcemia, and hyperuricemia. Once this occurs, this would rapidly progress to life-threatening complications, such as arrhythmia and renal failure (14). For the current case, when the patient was admitted, lactate dehydrogenase was 1,207 U/L (109-245 U/L), urea nitrogen was 15.71 mmol/L (3.20-7.10 mmol/L), creatinine (enzymatic method) was 133.6 μmol/L, calcium was 2.06 mmol/L (2.10-2.55 mmol/L), and creatine kinase was 261 U/L (38-174 U/L), indicating signs of cardiac and renal dysfunction prior to surgery, accompanied by electrolyte imbalance. In the later stage of hospitalization after the diagnosis of BL, the patient’s lactate dehydrogenase sharply increased to 3,411 U/L (109-245 U/L), accompanied by uric acid of 658.0 μmol/L (208.0-428.0 μmol/L), urea nitrogen of 29.27 mmol/L (2.90-8.20 mmol/L), creatine kinase isoenzyme CK-MB of 14.0 ng/ml (<6.6 ng/ml), and hypersensitive troponin I of 2,322.9 ng/L (<26.2 ng/L). Furthermore, severe cardiac and renal dysfunction, and hyperuricemia occurred, which highly suggested that the patient may have developed TLS.

The classic CHOP regimen (cyclophosphamide, doxorubicin, vincristine, and prednisone) combined with methotrexate has a high treatment failure rate (15). Subsequently, the CODOX-M/IVAC regimen proposed by Magrath et al. (cyclophosphamide, doxorubicin, vincristine, methotrexate, cyclophosphamide, cytarabine, and etoposide) presents with some advantages in improving the cure rate, but its bone marrow suppression and other toxic reactions are more severe (16, 17). Dunleavy et al. added rituximab on the basis of relevant research. In a prospective study, they used the EPOCH-R regimen (etoposide, doxorubicin and cyclophosphamide combined with vincristine, prednisone and rituximab) for adult BL (18), which was more effective for patients without bone marrow or CNS involvement. In addition, the study conducted by Barnes et al. confirmed that the combination of rituximab and CODOX-M/IVAC regimen can improve the progression-free survival (PFS) and overall survival (OS) of patients (19). This indicates that rituximab has certain advantages in combination therapy, but further and larger prospective studies are needed to confirm this. Unfortunately, for the current case, due to the worsening of the patient’s subsequent condition, further treatment was not possible.

In summary, BL is a relatively rare complication after liver transplantation, and early detection and treatment are crucial. For suspected BL, timely diagnostic methods, such as laboratory, imaging and pathology, should be applied to clarify the diagnosis. For advanced BL, special attention should be given in preventing the occurrence of TLS. Further research and exploration are needed to determine the optimal treatment plan for adult BL.

## Data Availability

The original contributions presented in the study are included in the article/supplementary material, Further inquiries can be directed to the corresponding author/s.
